# Association between dietary saturated fatty acid intake and rheumatoid arthritis: A cross-sectional study based on NHANES

**DOI:** 10.1097/MD.0000000000047214

**Published:** 2026-01-16

**Authors:** Jianyun Xu, Yangfan Wang, Qi Rao, Maosen Wang, Wei Yu, Chuhua Huang

**Affiliations:** a Qichun County People’s Hospital, Huanggang, Hubei Province, China.

**Keywords:** National Health and Nutrition Examination Survey, regression model, restricted cubic splines, rheumatoid arthritis, saturated fatty acid intake

## Abstract

This study aimed to investigate whether there is an association between saturated fatty acid (SFA) intake and the risk of rheumatoid arthritis (RA). A cross-sectional study was conducted on 15,885 participants from the National Health and Nutrition Examination Survey between 2003 and 2023. A multivariate logistic regression model was used to assess the relationship between SFA and RA. Additionally, receiver operating characteristic curves and sensitivity analyses were employed to evaluate the predictive ability and stability of the model. Furthermore, restricted cubic spline analysis was used to explore the nonlinear relationship between SFA and RA. Results showed a significant negative correlation between SFA intake and RA risk (odds ratio [OR] = 0.801, 95% confidence interval [CI]: 0.720–0.890, *P* < .001). After adjusting for confounding factors such as age, gender, race, education level, body mass index, poverty income ratio, smoking status, alcohol intake, and total energy intake, Model 2 (OR = 0.872; 95% CI: 0.775–0.981, *P* = .023), and Model 3 (OR = 0.798; 95% CI: 0.695–0.917, *P* = .002) showed that the same significant negative correlation between SFA intake and RA persisted. Receiver operating characteristic and sensitivity analyses confirmed the robustness of the results (area under the curve = 0.78). Restricted cubic spline analysis results suggested that there was a nonlinear relationship between SFA intake and RA risk (*P* for non-linearity = .0273). These results suggest that a low SFA intake may have a protective effect against RA and significantly reduce the risk of developing RA.

## 1. Introduction

Rheumatoid arthritis (RA), characterized as a chronic systemic autoimmune disorder, mainly impacts the synovial lining of the joints.^[1]^ The synovium becomes hyperplastic and infiltrated by inflammatory cells, resulting in the secretion of pro-inflammatory cytokines and the formation of pannus and new blood vessels. This process subsequently invades cartilage and bone tissue, ultimately resulting in severe joint damage and functional impairment.^[2]^ Since 1990, the annual global occurrence of RA has risen by roughly 8.2% each year, and with the intensifying aging of the population, RA has made a significant impact on global public health burden.^[3]^ Although the exact causes of RA remain unclear, recent research has begun to identify dietary factors that may contribute to its development.^[4]^ Relevant research indicated that inflammation plays a crucial role in the pathogenesis of RA.^[5]^ Inflammatory factors produced locally in the joints of RA patients, such as cytokines and chemokines, lead to synovial hyperplasia, pannus formation, and cartilage destruction.^[6]^

Fatty acids are an indispensable component of various tissues in the human body, playing crucial roles in maintaining tissue homeostasis, enhancing immune function, and regulating numerous metabolic processes.^[7]^ In recent years, the field of nutrition has delved deeply into the relationship between diet and human health, with a particular focus on the role of unsaturated fatty acids in RA becoming a research hotspot.^[8,9]^ However, saturated fatty acids (SFAs) have also garnered considerable attention and have been reported as one of the potential risk factors for the development of RA.^[10]^ A relevant study indicated that excessive SFA intake may exacerbate the inflammatory response in RA patients and potentially promote the process of muscle loss.^[11]^ Moreover, the dietary guidelines for RA issued by the American College of Rheumatology explicitly advise patients to restrict their SFA intake in order to maintain a healthy state.^[12]^ In summary, SFA intake is not only associated with health issues such as obesity and hyperlipidemia but may also significantly impacts the occurrence and progression of RA by affecting inflammatory responses.^[13]^

The National Health and Nutrition Examination Survey (NHANES) is a broad research program focused on providing a thorough assessment of the health and nutrition of both adults and children within the United States.^[14]^ The uniqueness of this survey is its integrated use of questionnaires and physical examinations, which enables the collection of demographic, socioeconomic, medical history, and other health-related information from participants. Based on RA-related data from the NHANES database for the years 2007 to 2023,^[15]^ the study investigated the association between SFA intake and the risk of RA using questionnaire data and dietary intake information.

## 2. Methods

### 2.1. Study population

NHANES is a carefully designed cross-sectional survey aimed at collecting comprehensive health and nutrition data for the U.S. population. The database employs a complex stratified, multi-level cluster sampling method to ensure a representative statistical sample of the entire U.S. population. The participants in NHANES submitted written consent forms, that received review and approval from the ethics review board at the National Center for Health Statistics, and adhered to the fundamental rules of the Declaration of Helsinki. This study employed NHANES data related to RA in the United States from 2007 to 2023 for subsequent data analysis.

**Figure 1.**
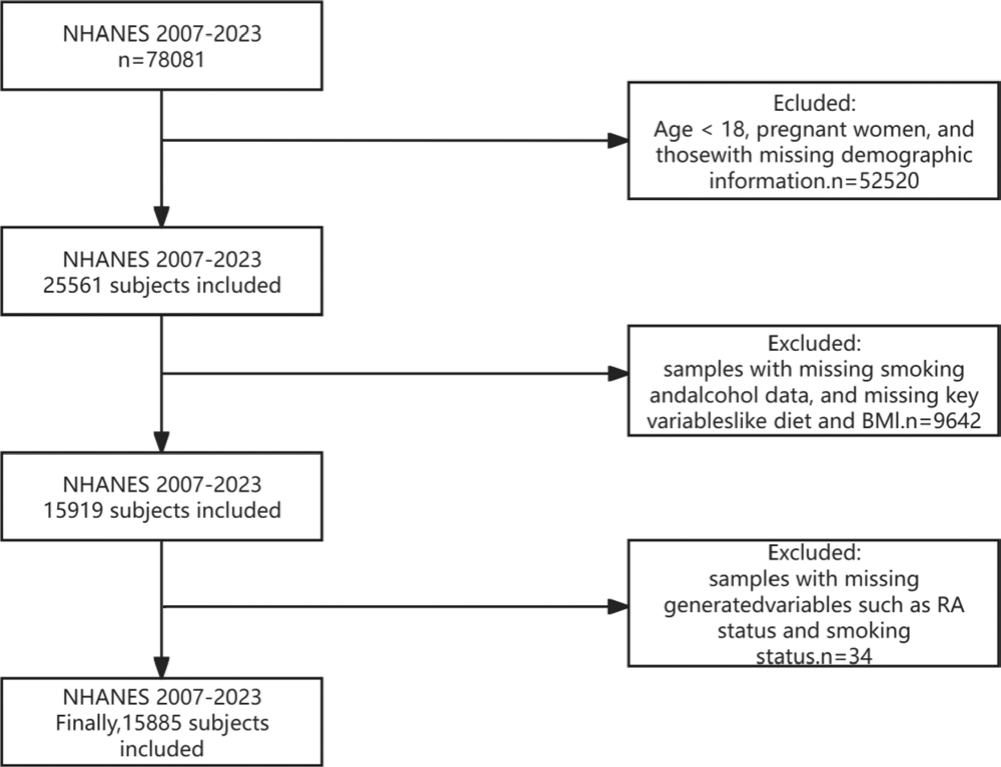
Flowchart of the selection process for RA-related participants in NHANES 2007 to 2023. NHANES = National Health and Nutrition Examination Survey, RA = rheumatoid arthritis.

The study included 78,081 participants from NHANES. We applied the following exclusion criteria (Fig. 1) Exclusion of participants under 18 years old, pregnant women, and those missing statistical information (N = 52,520); exclusion of participants missing data on smoking and alcohol consumption, as well as key variables such as diet and body mass index (BMI) (N = 9642); exclusion of participants missing derived variables such as RA status and smoking history (N = 34). The final study population consisted of 15,885 participants, comprising 2972 individuals diagnosed with RA and 12,913 control subjects without RA.

### 2.2. Assessment of saturated fatty acid and energy intake

SFA and energy intake were obtained from the 1st 24-hour dietary recall (SFA: DR1TSFAT, energy: DR1TKCAL), with specific values detailed in Table S1, Supplemental Digital Content, https://links.lww.com/MD/R149.^[16,17]^ The measurement tools and computational methodology for assessing participants’ dietary intake are described in detail within the survey protocol, accessible via the Centers for Disease Control and Prevention website (https://wwwn.cdc.gov/nchs/nhanes/tutorials/DietaryAnalyses.aspx). Furthermore, dietary nutrient intakes were calculated using the Food and Nutrition Database for Dietary Studies 5.0 (http://www.ars.usda.gov/ba/bhnrc/fsrg) standard of the Ministry of Agriculture.^[18]^ Based on the median SFA intake, the participants were divided into a low SFA group and a high SFA group.

### 2.3. Assessment of RA

In the NHANES questionnaires, participants will be asked a question regarding whether they have arthritis, with response options of “Yes,” “No,” “Refused,” and “Don’t know.” If a participant responds with “Yes,” he or she will then be proceed to a follow-up questionnaire about the type of arthritis, offering options such as “Osteoarthritis or degenerative arthritis,” “Rheumatoid arthritis,” “Psoriatic arthritis,” “Other,” “Refused,” and “Don’t know.” The participants who respond “Yes” to “Rheumatoid arthritis” will be identified as having RA.^[19]^

### 2.4. Covariates

The covariates in our study included a series of demographic and health-related variables, including age (18–30, 31–45, and >45), gender (male and female), race (White, Black, Mexican American, Hispanic, and other), education level (9–11th grade or no diploma for 12th grade, high school graduate or equivalent and college graduate or above), poverty income ratio (low, PIR < 1.3; medium, 1.3 ≤ PIR < 1.85; high, PIR ≥ 1.85),^[20]^ BMI (BMI < 25 kg/m², BMI ≥ 25 kg/m²),^[21]^ smoking status (smoker and nonsmoker), alcohol status (drinker and nondrinker), total SFA intake within 24 hours (g), and energy intake (kcal).

### 2.5. Statistical analysis

All data processing and analysis were conducted using R (v4.4.1; The R Foundation for Statistical Computing, Vienna, Austria). To conduct analysis of significant differences between RA and non-RA groups, we constructed a baseline table of covariates, which included categorical variables (such as age, gender and education level, etc) and continuous variables (such as DR1TSFAT). Using the R package “survey (v4.4-2),” we calculated the proportions (%) of each categorical variable among RA patients and non-RA participants, as well as the median, 1st quartile (Q1) and 3rd quartile (Q3) for continuous variables. All statistics, except for sample size, were adjusted for weighting. Differences between groups were assessed using Chi-square tests and Wilcoxon rank-sum tests, with statistical significance set at a *P*-value of <.05.

Using the R package ‘survey (v4.4-2)’, 3 logistic regression models were constructed to explore the association between SFA intake and RA. When building these 3 regression models, SFA intake was categorized into high and low groups based on a threshold of 19.94 g/d.^[18]^ Each model included different sets of confounding factors for analysis. Model 1 was constructed without considering any confounding factors. Model 2 incorporated variables for age, gender and race. Model 3, expanding on Model 2, further added variables for education level, BMI, PIR, smoking status, alcohol status, and energy intake. Additionally, we used the R package “ggplot2 (v3.5.1)” to create forest plots that displayed the impact of each variable in Model 3 on the risk of RA. We also used the R package “pROC (v1.18.5)” to generate receiver operating characteristic (ROC) curves to examine the predictive effectiveness of Model 3 for RA.

To assess the stability of RA risk analysis results across different thresholds of SFA intake, we conducted a sensitivity analysis. In this analysis, we established 3 distinct 24-hour SFA intake thresholds: 15 g, 20 g, and 25 g. For each threshold, the odds ratios (ORs) and 95% confidence intervals (CIs) were calculated. Furthermore, we utilized restricted cubic spline (RCS) analysis to investigate the nonlinear relationship between SFA intake and RA prevalence. The fitting results of the nonlinear model were visualized with the R package “ggplot2 (v3.5.1).”

## 3. Results

### 3.1. Baseline characteristics

As shown in Table 1, the significant differences existed (*P* < .05) between RA patients and controls with respect to age, gender, race, BMI, PIR, smoking status, alcohol status, and SFA intake (DR1TSFAT). The study sample consisted of 15,885 participants, of whom 75.1% were male and 24.9% were female. Among the RA patients, 83.7% of the participants were male, 79% were White, 3.7% were Mexican American, 8.9% were Black, and 3.2% were Hispanic. The median SFA intake for RA participants was 26.87 g/d, while the median SFA intake for non-RA participants was 25.7 g/d. Compared to non-RA participants, those with RA tended to be predominantly male. Regarding socioeconomic status, RA participants tended to have lower education levels and lower incomes. With respect to lifestyle habits, RA participants had higher proportions of smoking and alcohol status. Additionally, RA participants had higher BMI and greater energy intake (Table 1).

**Table 1:** Baseline characteristic table for RA and Non-RA participants.

Characteristic	Non-RA (N = 12,913)	RA (N = 2972)	*P*-value
Age (%)			<.001
18–30	4519 (35)	202 (6.8)	
31–45	5023 (38.9)	779 (26.2)	
>45	3371 (26.1)	1991 (67)	
Gender (%)			<.001
Female	4326 (33.5)	484 (16.3)	
Male	8587 (66.5)	2488 (83.7)	
Race (%)			<.001
Mexican American	1072 (8.3)	110 (3.7)	
Hispanic	826 (6.4)	95 (3.2)	
White	8561 (66.3)	2348 (79)	
Black	1395 (10.8)	265 (8.9)	
Other	1059 (8.2)	154 (5.2)	
Education level (%)			.4159
9–11th grade or 12th grade without diploma	1085 (8.4)	279 (9.4)	
High school graduate or equivalent	2854 (22.1)	636 (21.4)	
College graduate or above	8974 (69.5)	2057 (69.2)	
PIR (%)			.0376
<1.3	2776 (21.4)	568 (19.1)	
1.3–1.85	1498 (11.6)	312 (10.5)	
≥1.85	8639 (67)	2092 (70.4)	
BMI (%)			<.001
<25	4106 (31.8)	577 (19.4)	
≥25	8807 (68.2)	2395 (80.6)	
Smoking status (%)			<.001
Non-smoker	7360 (57)	1195 (40.2)	
Smoker	5553 (43)	1777 (59.8)	
Alcohol status (%)			<.001
Non-drinker	3461 (26.8)	966 (32.5)	
Drinker	9452 (73.2)	2006 (67.5)	
DR1TSFAT (Median (Q1, Q3))	25.7 (16.68, 37.49)	26.87 (18.18, 38.83)	.0003
DR1TKCAL (Median (Q1, Q3))	2172 (1628, 2856)	2201 (1651, 2860)	.2046

BMI = body mass index, PIR = poverty income ratio, RA = rheumatoid arthritis, SFA = saturated fatty acid.

### 3.2. Association between SFA intake and RA

In the univariate regression analysis (Model 1), when SFA intake was converted into a categorical variable (high and low SFA intake groups), a significant negative correlation was found between the low SFA intake group (SFA_Intake_GroupLow) and the risk of RA (*P* < .05). The OR for the low SFA intake group was 0.801, with a 95% CI of 0.720 to 0.890, indicating that a lower intake of SFA significantly reduced the risk of RA (Table 2).

**Table 2:** The results of the univariate regression analysis for SFA intake (Model 1).

Variable	OR	95% CI (lower–upper)	*P*-value
(Intercept)	0.222	(0.206–0.239)	<2e-16
SFA_intake_group_low	0.801	(0.720–0.890)	.000059

CI = confidence interval, OR = odds ratio, SFA = saturated fatty acid.

After incorporating basic covariates such as age, gender and race into Model 2, SFA intake remained a significant factor for the risk of RA. The OR was 0.872, with a 95% CI of 0.775 to 0.981 and a *P*-value of .023. This still showed a significant association between low SFA intake and the RA risk, suggesting that the impact of SFA intake on RA persisted even after adjusting for other factors (Table 3).

**Table 3:** The results of the regression analysis after including covariates (age, gender, and race) in Model 2.

Variable	OR	95% CI (lower–upper)	*P*-value
(Intercept)	0.025	(0.019–0.031)	<2e-16
SFA_intake_group_low	0.872	(0.775–0.981)	.023
Gender (female)	1.223	(1.022–1.464)	.029
Age_group (31-45)	3.437	(2.801–4.216)	<2e-16
Age_group (>45)	13.661	(11.068–16.861)	<2e-16
Race (Hispanic)	1.003	(0.796–1.263)	.982
Race (White)	1.789	(1.511–2.119)	<2e-16
Race (Black)	1.544	(1.276–1.867)	<0.001
Race (Other)	1.151	(0.882–1.502)	.297

CI = confidence interval, OR = odds ratio, SFA = saturated fatty acid.

**Figure 2.**
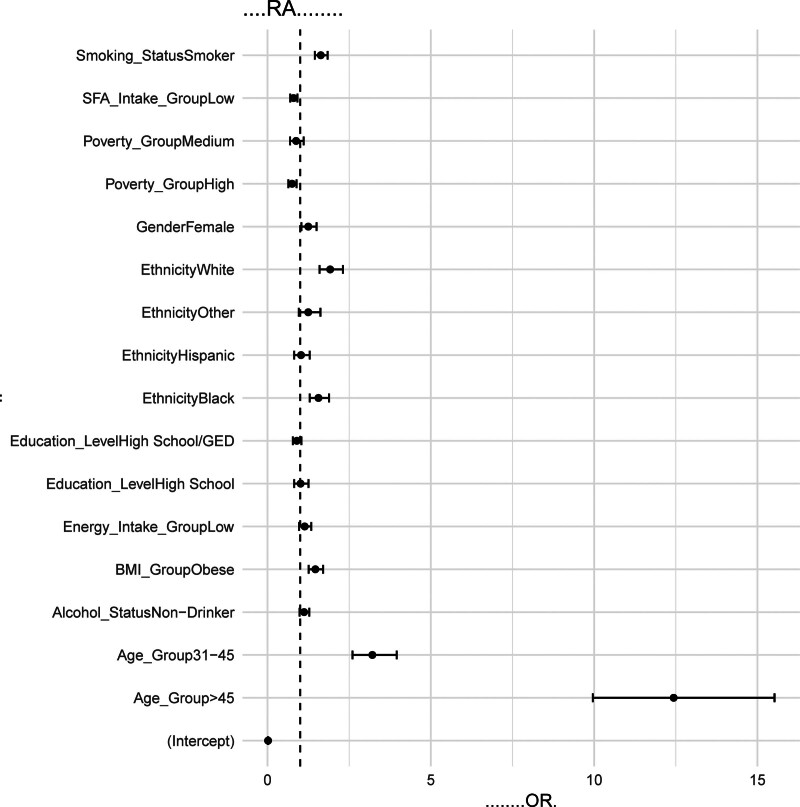
Association analysis between low SFA intake and the risk of RA. It displays a forest plot for a multivariate regression analysis (Model 3) that includes the covariates of age, gender, race, education level, smoking status, alcohol status, PIR, BMI and energy intake. The ORs and the 95% CIs are shown in the figure, with a vertical dashed line indicating OR = 1. BMI = body mass index, CI = confidence interval, OR = odds ratio, PIR = poverty income ratio, RA = rheumatoid arthritis, SFA = saturated fatty acid.

In Model 3, after incorporating all covariates, the impact of SFA intake on RA remained significant. The OR was 0.798, with a 95% CI of 0.695 to 0.917 and a *P*-value of .002, which continued to indicate a significant association between lower SFA intake and RA risk. The exposure factor (low SFA intake) maintained *P*-values <.05 across all 3 models, suggesting that the effect of SFA intake on RA was robust and not confounded by other covariates (Table 4, Fig. 2).

**Table 4:** The results of the regression analysis after including all covariates in Model 3.

Variable	OR	95% CI (lower–upper)	*P*-value
(Intercept)	0.017	(0.013–0.023)	<2e-16
SFA_intake_group_low	0.798	(0.695–0.917)	.002
Gender (female)	1.247	(1.036–1.501)	.020
Age_group (31–45)	3.210	(2.603–3.958)	<2e-16
Age_group (>45)	12.435	(9.960–15.524)	<2e-16
Race (Hispanic)	1.026	(0.816–1.291)	.824
Race (White)	1.919	(1.594–2.310)	<2e-16
Race (Black)	1.559	(1.291–1.883)	<0.001
Race (Other)	1.246	(0.959–1.620)	.099
Education_level (high school)	1.012	(0.817–1.254)	.911
Education_level (high school/GED)	0.897	(0.776–1.037)	.141
PIR_group (medium)	0.875	(0.691–1.107)	.263
PIR_group (high)	0.753	(0.639–0.889)	.001
BMI_group (obese)	1.465	(1.261–1.701)	<.001
Smoking_status (smoker)	1.631	(1.447–1.840)	<.001
Calorie_intake_group (low)	1.138	(0.968–1.338)	.116

CI = confidence interval, OR = odds ratio, SFA = saturated fatty acid.

In summary, participants with lower SFA intake showed a negative correlation with the risk of RA across multiple regression models. Particularly after incorporating all covariates, low SFA intake remained significantly associated with a reduced risk of RA. This suggested that reducing SFA intake might have potential benefits for the prevention of RA.

### 3.3. ROC curve

**Figure 3.**
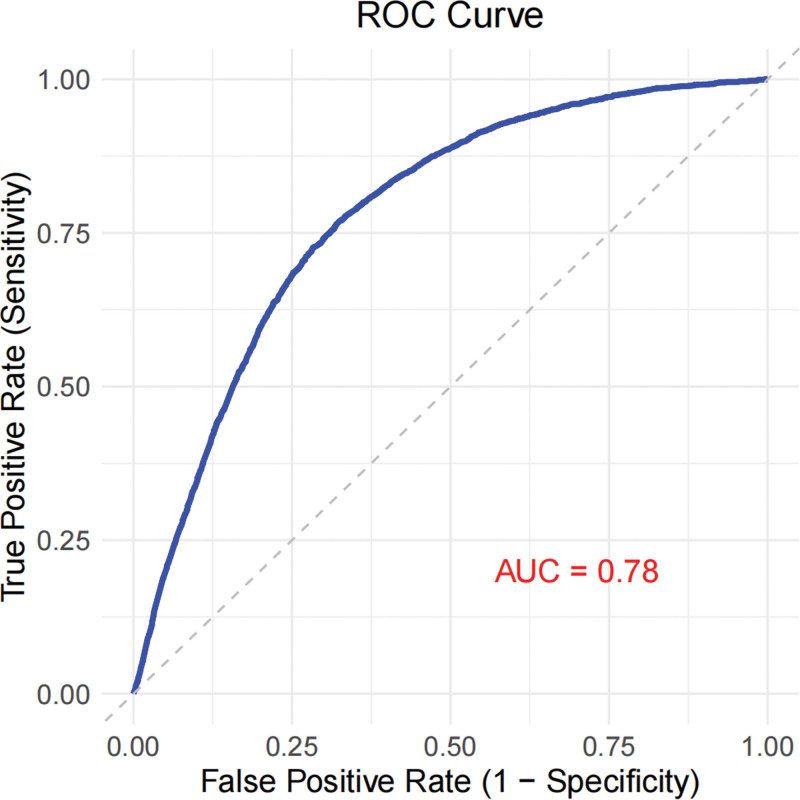
ROC curve for the multivariate regression analysis (Model 3). ROC = receiver operating characteristic.

The ROC curve result indicated that the area under the curve value of the multivariate regression analysis (Model 3), which included the exposure factor (SFA_Intake_GroupLow), was 0.78 when predicting the risk of RA. This suggested that the multivariate regression model had a certain diagnostic effect for RA (Fig. 3).

### 3.4. Sensitivity analysis

**Figure 4.**
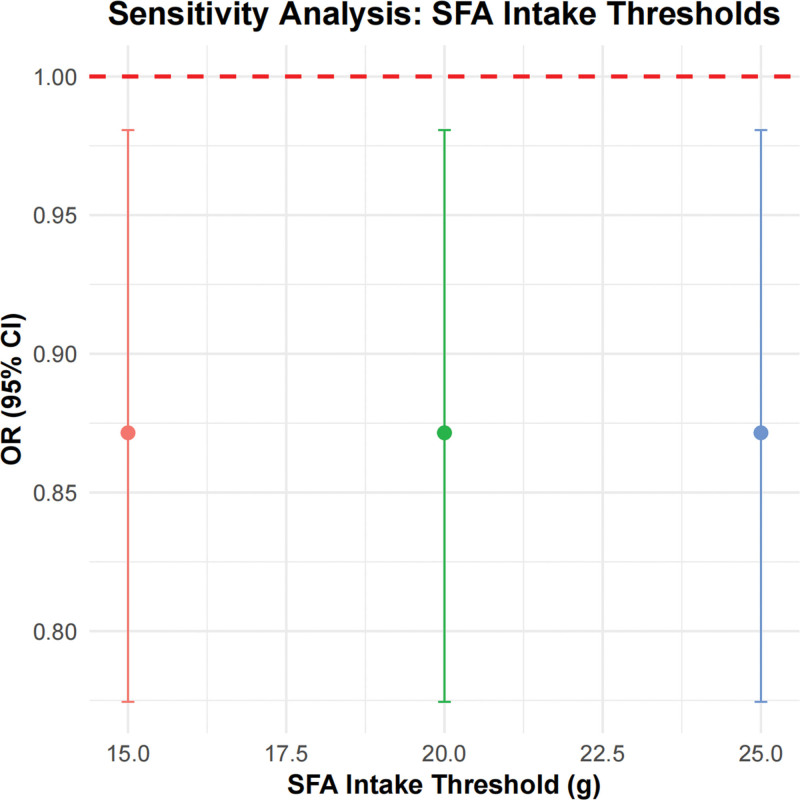
Sensitivity analysis of RA risk at different SFA intake thresholds. The figure shows the ORs and the 95% CIs for different SFA intake thresholds. Each point represents the OR at a specific threshold, with error bars indicating the 95% CI. A red dashed line represents the reference line where OR = 1. CI = confidence interval, OR = odds ratio, PIR = poverty income ratio, RA = rheumatoid arthritis, SFA = saturated fatty acid.

According to the sensitivity analysis result, when the daily SFA intake thresholds were set at 15 g, 20 g, and 25 g, the OR consistently remained at 0.87, with 95% CI of 0.77 to 0.98. This consistency across different thresholds indicated that the OR values did not vary with increasing SFA intake thresholds, and both the OR values and 95% CIs were below 1. These findings suggested that lower SFA intake might have a protective effect against the RA development, potentially reducing the risk of RA (Fig. 4).

### 3.5. RCS analysis

**Figure 5.**
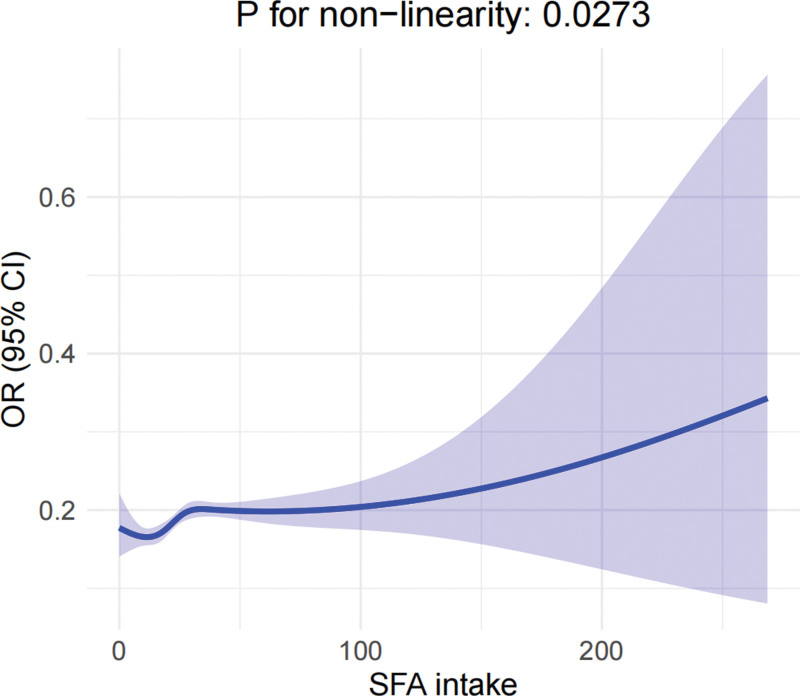
The RCS analysis of SFA intake and RA risk. The figure displays the nonlinear relationship between SFA intake and the risk of RA. The blue curve represents the predicted ORs for RA risk based on a natural spline model (degrees of freedom = 4), and the shaded area around the curve indicates the 95% CIs. CI = confidence interval, OR = odds ratio, RA = rheumatoid arthritis, RCS = restricted cubic spline, SFA = saturated fatty acid.

The RCS analysis showed a positive correlation between SFA intake and RA risk. At lower levels of SFA intake, the risk of RA was lower, and as SFA intake increased, the risk of RA showed a gradual upward trend. The result also suggested that as SFA intake increased, the 95% CIs also widened, indicating greater uncertainty in the model at higher levels of SFA intake (Fig. 5).

## 4. Discussion

This cross-sectional study, which encompassed 15,885 U.S. participants, we extracted data from the NHANES database for the period 2007 to 2023. The data involved age, gender, race, education level, PIR, BMI, smoking status, alcohol status, SFA intake, and energy intake. Our objective was to investigate the association between SFA intake and RA. Our results suggested that there was a significant difference in SFA intake between the RA and non-RA groups and lower SFA intake might reduce the risk of RA.

The development of RA involves complex mechanisms. The primary pathological characteristics include persistent synovitis, a disturbance in osteoblast–osteoclast homeostasis, and dysfunction of immune cells.^[19,22]^ The inflammatory process of arthritis in RA is initiated and perpetuated through a network of interactions involving fibroblasts, osteoclasts, B cells, T cells, neutrophils, and macrophages.^[23]^ Additionally, the onset of RA is also influenced by cytokines, including TNF-α, IL-6, and IFN-γ.^[24,25]^

SFAs are predominantly derived from animal fats and specific plant oils. High intake of SFAs may raise the risk of chronic conditions like heart disease, obesity, and inflammation.^[22,26,27]^ SFAs may also negatively impact immune responses. Some studies have shown that SFAs can influence the activity and function of immune cells such as T cells, B cells, and macrophages.^[28]^ These immune cells are critical to the pathogenesis of RA, and their abnormal activation and proliferation can lead to joint inflammation and damage.^[29]^ Therefore, excessive SFA intake may increase the risk of RA by affecting the function of the immune system. Additionally, SFAs may indirectly influence the risk of RA by impacting metabolic processes within the body, such as lipid metabolism and glucose metabolism.^[30,31]^ Metabolic abnormalities may exacerbate inflammatory responses within the body, thereby increasing the risk of developing RA. Additionally, such abnormalities can also influence the effectiveness and safety of medications, making the treatment of RA more challenging.^[32]^

A study had shown that the Mediterranean diet, as a dietary regimen for RA patients, was closely associated with reduced inflammation levels.^[27]^ This may be directly related to the higher intake of ω-3 polyunsaturated fatty acids in the Mediterranean diet, as these fatty acids demonstrate anti-inflammatory characteristics and can decrease the frequency of nonsteroidal anti-inflammatory drug use among RA patients.^[33,34]^ Furthermore, the higher intake of fruit juices in the Mediterranean diet also confers significant benefits to RA patients. This is because fruit juices contain high levels of polyphenols, which also have anti-inflammatory properties.^[35,36]^ While these study results did not directly prove that low SFA intake can reduce the risk of RA, they suggested that healthy dietary habits, including lower SFA intake and higher intake of unsaturated fatty acids, may aid in the prevention of RA.^[37]^ These study results further confirmed that reducing the SFA intake was correlated with a lower risk of RA.

This study also had some limitations. First, since most of the covariables in our study were based on surveys, there was potential for subjective information bias, which might have affected the accurate estimation of SFA intake. Second, the study investigated the association between SFA intake and the risk of RA. Self-reported RA diagnoses, along with the characteristic long-term development of chronic RA, might have introduced data bias. Additionally, baseline data were unlikely to fully capture changes in participants’ dietary habits over the course of the study, which could have influenced the accuracy of the results. Our study did not examine the potential associations between specific SFA subtypes and RA risk, which will be a primary focus of our future research. Finally, due to the cross-sectional nature of this study and the lack of a time series component, it was not possible to perform further analysis to explore the causal relationship between SFA intake and RA.

## 5. Conclusion

This study utilized NHANES data and employed statistical methods such as regression analysis, sensitivity analysis and RCS analysis to reveal an association between SFA intake and RA. The findings indicated that as SFA intake increased, the risk of RA showed a gradual upward trend. Lower SFA intake seemed to offer protection against the development of RA, significantly lowering the risk of its occurrence.

## Acknowledgments

We expressed our gratitude to all individuals who participated in the study.

## Author contributions

**Conceptualization:** Jianyun Xu.

**Data curation:** Maosen Wang.

**Formal analysis:** Yangfan Wang.

**Investigation:** Qi Rao, Wei Yu.

**Methodology:** Jianyun Xu, Chuhua Huang.

**Resources:** Chuhua Huang.

**Software:** Yangfan Wang.

**Supervision:** Qi Rao.

**Validation:** Maosen Wang.

**Visualization:** Wei Yu.

**Writing – original draft:** Jianyun Xu.

**Writing – review & editing:** Chuhua Huang.

## Supplementary Material


